# Hepatic Haemangioma Masquerading as the Gallbladder in a Case of Gallbladder Agenesis: A Case Report and Literature Review

**DOI:** 10.1155/2010/971609

**Published:** 2010-07-19

**Authors:** James A. Stephenson, Michael Norwood, Dhya Al-Leswas, Omer Al-Taan, Richard Beable, David M. Lloyd, Ashley R. Dennison

**Affiliations:** ^1^Department of Hepatobiliary and Pancreatic Surgery, Leicester General Hospital, Gwendolen Road, Leicester LE5 4PW, UK; ^2^Department of Radiology, University Hospitals of Leicester NHS Trust, Gwendolen Road, Leicester LE5 4PW, UK

## Abstract

Gallbladder agenesis is uncommon. In contrast, liver haemangiomas are the most common type of benign liver lesions. We describe the first documented case of gallbladder agenesis where the clinical presentation was consistent with biliary colic, and radiological investigation suggested the presence of gallstones. Subsequent operative findings revealed a solitary haemangioma of the liver sited in the normal position of the gallbladder fossa but with absence of the gallbladder. It is important that clinicians should keep gallbladder agenesis in mind when the gallbladder appears abnormal on preoperative imaging studies and cannot be found at laparoscopy. As symptoms will improve in 98% of cases, it is very important to avoid unnecessary intervention in patients who have a negative laparoscopy. The clinical presentation, investigations, and operative findings are discussed with a review of other relevant reported cases in the literature.

## 1. Introduction

Gallbladder agenesis has been reported in the medical literature but is uncommon. 

 It is thought to occur in approximately 1 in 6,000 live births [1]. In contrast, liver haemangiomas are the most common type of benign liver lesions. We describe the first documented case of gallbladder agenesis where the clinical presentation was consistent with biliary colic, and radiological investigation suggested the presence of gallstones within a collapsed, fibrosed gallbladder. Subsequent operative findings revealed a solitary haemangioma of the liver sited in the normal position of the gallbladder fossa but with absence of the gallbladder.

## 2. Case Presentation

A 23-year-old female presented with intermittent episodes of nausea, vomiting, right upper quadrant abdominal pain, and occasional diarrhoea. Clinical examination was unremarkable. Routine haematological and biochemical investigations including full blood count profile, c-reactive protein, liver function tests (including alanine aminotransferase, total bilirubin, *γ*-glutamyltransferase, and alkaline phosphatase), and amylase were unremarkable. Stool culture demonstrated no abnormality, and faecal elastase was within the normal range. Abdominal ultrasonography suggested a contracted gallbladder containing gallstones.

**Figure 1:**
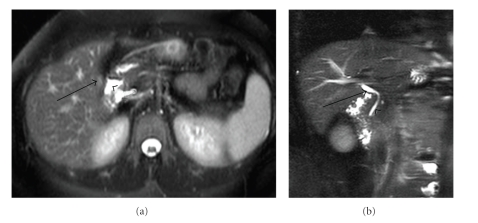
(a) Axial T2-weighted half-fourier acquisition single-shot turbo spin echo (HASTE) MRI image demonstrating a faint area of high signal in the area of the gallbladder fossa (arrow) adjacent to the second part of the duodenum (arrowhead). The radiological appearances are suggestive of a small contracted gallbladder. Although with the absence of a recognisable cystic duct, a surface haemangioma is a possible differential diagnosis. (b) T2 weighted coronal MRI image with fat suppression demonstrating a normal calibre extrahepatic biliary ducts (arrow). Note the absence of any cystic duct. Normal appearance of the pancreatic duct (arrowhead).

On clinical review following the ultrasound examination, the patient's pain had improved but the vomiting persisted. Due to the continued vomiting, nonclassic symptoms of gallstones disease, and absence of dyspeptic symptoms, magnetic resonance cholangiopancreatography (MRCP) was performed. The MRCP report commented that the gallbladder was difficult to identify but appeared to be contracted (Figure 1(a)). The common bile duct was not dilated, and there were no filling defects (Figure 1(b)). 

**Figure 2:**
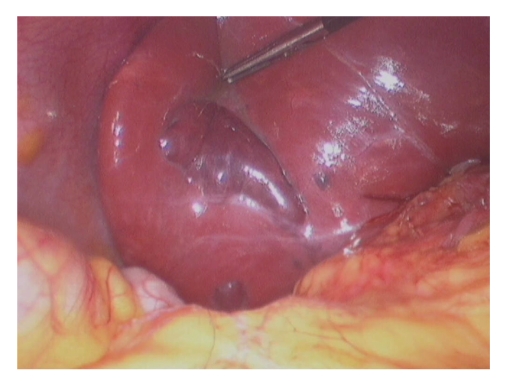
Laparoscopic image demonstrating the Hepatic Haemangioma in the gallbladder fossa.

At laparoscopy, the gallbladder and cystic duct could not be identified, either by direct vision or by the use of the intraoperative ultrasound probe. A pear-shaped haemangioma was present on the surface of segment 6 of the liver within the gallbladder fossa mimicking the gallbladder (Figure 2). No further procedure was undertaken. Further imaging was requested postoperatively to locate a potential ectopic gallbladder. 

The patient recovered well from surgery, and her symptoms resolved. Subsequently repeated MRCP, confirmed the absence of the gallbladder, a normal common bile duct and no change in the haemangioma. The patient remained well and symptom free 2 years later.

## 3. Discussion

Congenital anomalies of the gallbladder are numerous, and in addition to agenesis, include location in an abnormal site (ectopic gallbladder) hypoplasia, diverticula, duplication, heterotrophic tissue, and septation defects.

Agenesis of the gallbladder (AG) is a rare congenital anomaly of the biliary system and was first described by Lemary in 1701 [2]. The average incidence of gallbladder agenesis at birth is about 0.02% [2], or 1 in 6000 live births. AG has been observed in both children and adults, with a median age of 46 at the time of diagnosis [3]. Clinical studies report a female-male ratio of 3 : 1, while autopsy studies interestingly report an equal gender incidence [1].

AG is due to a fault in embryological development of the gallbladder. Approximately 70% of cases are thought to be isolated anomalies, and 9% are associated with biliary atresia. The gallbladder is developed by the 4th week of gestation from the pars cystica (caudal portion) of the anterior diverticulum of the primitive gut. The pars cystica originates from the anterior duodenum becoming the common bile duct after rotation of the duodenum. The gallbladder is initially a hollow cord as the hollow portion of the pars cystica is obliterated by epithelial proliferation. Essentially, AG is an anomaly of the development of vessels located on each side of the gallbladder bud (sinus venosus cordis, omphaloenteric, and umbilical veins) [4] which may explain the association of this anomaly in 21% of cases with cardiac, vascular, gastro-intestinal, and abdominal wall malformations observed in the multiple fetal anomaly group [5]. AG may be inherited, with several familial cases observed, including across two generations [6–11]. These series suggest a non-sex-linked inheritance pattern with variable penetration.

Bennion et al. [3] suggested a classification system for AG based on a review of cases worldwide. They identified three groups: (1) multiple fetal anomalies, the most common malformations being cardiovascular, followed by gastrointestinal and genitourinary, (2) asymptomatic cases, in which agenesis of the gallbladder was found at autopsy or laparotomy for another reason and, in some cases, there was a familial association, and (3) symptomatic cases, with the most common associated symptoms being biliary colic (54%), dyspepsia (34%), and jaundice (27%) [3, 12].

The presenting symptoms of agenesis of the gallbladder are compatible with hepatobiliary dysfunction such as right upper quadrant pain, nausea, vomiting, abnormal liver function, and jaundice, and these symptoms are found in 40% of cases [13]. It is believed that these symptoms are a consequence of biliary dyskinesia, but 98% of patients report resolution of symptoms postoperatively where no formal procedure was performed other than identification of an absent gallbladder. This was the situation with our patient who did improve significantly following surgical exploration, but why this occurs remains unclear.

The majority of cases in the literature have been diagnosed at laparotomy, but with the increasing use of laparoscopic surgery, case reports describing the diagnosis of gallbladder agenesis at laparoscopy are common. Even with advances in imaging modalities, radiology reports are still frequently unable to be dogmatic, particularly in respect of anatomical findings, and USS often reveals what is thought to be a small, contracted gallbladder.

Gallbladder agenesis encountered during a surgical procedure is almost always unexpected and usually presents the clinician with a surgical dilemma. Routine performance of laparoscopic cholecystectomy has produced changes in technique, and the more limited dissection and lack of tactile feedback give less information about the anatomical relationships. This often prompts the conversion to an open procedure if a gallbladder cannot be found [14]. Frey emphasized the need for a thorough surgical exploration to exclude an ectopic gallbladder prior to being able to confidently confirm the diagnosis of gallbladder agenesis [15]. Recognised ectopic locations are intrahepatic, left-sided, between the leaves of the lesser omentum, retroperitoneal, retrohepatic, within the falciform ligament, retroduodenal, and retropancreatic [3, 14–16]. Unfortunately, the wide distribution of these possible abnormal sites means that considerable open dissection and consequent morbidity would occur if an attempt was made to fulfill Frey's criteria rigorously. A potentially less invasive method would be to use intraoperative ultrasonography (as was used in this case), which is able to give extremely clear images of all the potential ectopic sites.

In cases reported in the literature, intraoperative cholangiogram is felt to be essential to exclude or confirm the presence of common bile duct stones and to complete the diagnosis. Some groups recommend routine common bile duct exploration when its diameter is increased or a common bile duct stone has been found [3, 14, 16–18]. We elected not to perform an intraoperative cholangiogram or to explore the common bile duct because of reports of iatrogenic common bile duct injury produced during the search for an absent gallbladder. In addition, the preoperative MRCP images (which were reviewed intraoperatively) were of good quality, and we could not identify any filling defect suggesting ductal stones. If Frey's criteria are not fulfilled intra-operatively, other authors advocate post-operative CT imaging to search for a potential ectopic gallbladder and confirm the diagnosis of gallbladder agenesis [19].

## 4. Conclusion

It can be extremely difficult to diagnose agenesis of the gallbladder preoperatively using routine ultrasound examination. Consequently, in this era of minimally invasive surgery, it is important that clinicians should keep this rare condition in mind when the gallbladder appears abnormal on preoperative imaging studies and cannot be found at laparoscopy. As symptoms will improve in 98% of cases, it is very important to avoid unnecessary intervention in patients who have a negative laparoscopy. 

Laparoscopic exploration combined with laparoscopic ultrasonography and potentially post-operative CT or MRI is helpful in establishing a definitive diagnosis of agenesis of the gallbladder. They are also to refute a putative diagnosis of ectopic gallbladder, this approach will reduce the morbidity associated with more invasive surgery. When intra-operative imaging is able to exclude an ectopic location of the gallbladder and a diagnosis of gallbladder agenesis is established, then pain will resolve in almost all cases and further investigation will be difficult to justify.
